# Barriers to the implementation of mobile phone reminders in pediatric HIV care: a pre-trial analysis of the Cameroonian MORE CARE study

**DOI:** 10.1186/s12913-014-0523-3

**Published:** 2014-10-26

**Authors:** Jean Joel R Bigna, Jean Jacques N Noubiap, Claudia S Plottel, Charles Kouanfack, Sinata Koulla-Shiro

**Affiliations:** Faculty of Medicine and Biomedical Sciences, University of Yaoundé 1, Yaoundé, Cameroon; Faculty of Medicine, University of Montpellier 1, Montpellier, France; Preventing Mother to Child Transmission Unit, Goulfey District Hospital, Goulfey, Cameroon; Internal Medicine Unit, Edéa Regional Hospital, Edéa, Cameroon; Department of Medicine, New York University Langone Medical Center, New York, USA; Department of Medicine, Division of Translational Medicine, New York University School of Medicine, New York, USA; Accredited Treatment Centre, Yaoundé Central Hospital, Yaoundé, Cameroon; Infectious Disease Unit, Yaoundé Central Hospital, Yaoundé, Cameroon

**Keywords:** MORE CARE, Mobile phone, Telecare, Telemedicine, EHealth, Obstacles, mHealth, Literacy, HIV, Caregivers in pediatric HIV

## Abstract

**Background:**

Mobile health (mhealth) has emerged as a powerful resource in the medical armamentarium against human immunodeficiency virus (HIV) infection. We sought to determine among adult caregivers of HIV-exposed/infected children; the extent of mobile phone ownership, the ability to communicate in Cameroon’s national official languages (NOL), and the refusal to receive such reminders.

**Methods:**

We conducted a pre-trial analysis of potentials participants of the MORE CARE trial. MORE CARE took place from January through March 2013 in three geographic locations in Cameroon. We included caregivers aged 18 years or older. Written communication was assessed by the ability to read and understand information presented in the consent form. Verbal communication was assessed during a two-way conversation and in a discussion about HIV infection. A question about mobile phone ownership and another about refusal to receive reminders via mobile phone were phrased to allow “Yes” or “No” as the only possible reply. A *p* <0.05 was considered statistically significant.

**Results:**

We enrolled 301 caregivers of HIV-exposed/infected children from rural (n = 119), semi-urban (n = 142) and urban (n = 40) areas of Cameroon. The mean caregiver age was 42.9 years (SD 13.4) and 85% were women. A fifth of our study population overall had at least one of the three obstacles to mobile phone reminders. By region, 39.5% in rural, 6.3% in semi-urban, and 7.5% in urban setting had at least one obstacle, with significant differences between the rural and urban settings (*p*<0.001) and the rural and semi-urban settings (*p*<0.001). The acceptability of SMS was 96.3% and of mobile phone calls 96% (*p* = 0.054). The ability to communicate in NOL orally was 89.7% and 84.4% in writing (*p* = 0.052). Mobile phone ownership (*p*<0.001; *p* = 0.03) and the ability to communicate in an NOL orally (*p*<0.001; *p* = 0.002) or in writing (both *p*<0.001), were significantly lower in rural compared to semi-urban and urban settings respectively.

**Conclusions:**

The use of mHealth was limited in about one fifth of our population. The greatest obstacle was the inability to use oral or written NOL, followed by non-ownership of a mobile phone. These impediments were higher in a rural setting as compared to urban or semi-urban areas.

## Background

The Global Observatory for eHealth defines mobile health (mHealth) as medical and public health practice supported by mobile devices, such as mobile phones, patient monitoring devices, personal digital assistants, and other wireless devices [1]. The United Nations Joint Program on HIV/AIDS (UNAIDS) encourages the use of mHealth in addressing human immunodeficiency virus (HIV) related illnesses and treatments in resource-limited settings [2,3]. According to the International Telecommunications Union, the number of mobile-broadband subscriptions has reached 2.3 billion worldwide in 2014, with 55% of subscriptions based in developing countries. Africa leads in mobile-broadband growth [4]. MHealth is increasingly recognized as an effective adjunct to HIV control measures [5,6]. Cameroon, a sub-Saharan African country, has seen the proportion of mobile phone users increase rapidly [4,7] as elsewhere in Africa [8], with 60% of the Cameroonian population owning a mobile phone in 2012 [9]. The use of mobile phones has shown effectiveness in health related behavior change, in screening campaigns, and as a supportive tool in treatment, diagnosis, and data collection [3,6,10–23]. Challenges to the implementation of mHealth include lack of mobile phone ownership and an inability to communicate in national official languages (NOL) both via mobile phone call (oral) and mobile phone text message (SMS) (written). In 2011, about two-third of the adult population in Cameroon were literate [8]. An individual’s unwillingness to use a mobile phone for health-related communications represents another barrier to acceptance of mHealth. In certain resource-limited settings adherence to mHealth has been shown to be high, despite potential obstacles [6,24–26].

Notwithstanding the proven benefits of the use of mobile phones in the fight against HIV, challenges to implementation remain. Before adopting mobile technologies into the clinical arena, it is imperative to know exactly what e-health architecture to employ within a health system [18]. In Cameroon, the challenges that need to be addressed to effectively and efficiently implement SMS and mobile phone calls in pediatric HIV care remain undefined. In the recently reported MORE CARE (Mobile Reminders for Cameroonian children Requiring HIV treatment) study, we investigated the efficacy and efficiency of mobile phone appointment reminders on the attendance of HIV-exposed or infected children at their previously scheduled follow-up medical appointments [27]. The aim of the present study was to evaluate three factors as potential obstacles to the implementation of using mobile phones (SMS and calls) as medical appointment reminder tools for HIV-exposed and HIV-infected children in Cameroon. More specifically, we sought to investigate the extent of mobile phone non-ownership, the inability to communicate in a NOL, and the refusal to receive mobile phone reminders as variables influencing the acceptance of mobile phones as part of mHealth.

## Methods

### Ethical consideration

The study was approved by the Faculty of Medicine and Biomedical Sciences, University of Yaoundé 1. We obtained ethical clearance from the National Research Ethics Committee for Human Health of Cameroon (Ethical approval N° 2013/03/232/L/CNERSH/SP). All patients included in the study gave their verbal and written consent.

### Study design

This is a pre-trial cross-sectional study of adults included in the MORE CARE trial and addresses mobile phone ownership, an ability to communicate in NOL, and a willingness to receive health-related mobile phone appointment reminders. MORE CARE has been described in detail previously [23,27].

### Settings and participants

Potential participants in the MORE CARE study were enrolled in an urban, a semi-urban, and a rural setting at the Essos National Hospital Insurance Fund, the Kousséri Annex Regional Hospital, and the Goulfey District Hospital respectively [23,27]. Adults aged 18 years or older who accompanied a child to a medical appointment for HIV-related care were invited to enroll in the MORE CARE trial. Only one caregiver present at the time of recruitment was identified for each child. Although that adult caregiver was responsible for ensuring the child’s attendance at their next scheduled medical appointment, we did not require that the adult accompanying the child at that follow-up visit be the same adult since MORE CARE was designed to examine each child’s presence or absence at their follow-up visit for HIV care.

### Appointment reminders in the MORE CARE study

We briefly report here the MORE CARE mHealth appointment reminder methods, which are further detailed in the published study protocol [27]. The text message sent to caregiver participants was in French or in English (national official languages in Cameroon), and was edited manually. It included the physician's name and the date, time, and location of the appointment. In order to maintain confidentiality and to protect caregiver and patient privacy, the message did not contain information concerning the health status and name of the child, nor the name of the accompanist. The message was sent and each voice phone call placed 48 to 72 hours before the previously scheduled appointment. The content of phone calls was the same as that of the SMS. The communication during phone call was in English or in French, based on the caregiver’s stated preference.

### Variables

We collected the age and sex of caregivers and of the children they accompanied. Obstacles to mHealth appointment reminders investigated were:

1. The non-possession of mobile phone; assessed by asking a potential participant the question, “Do you own a mobile phone?” The answer could only be “Yes” or “No”.
2. The inability to understand and read English or French (SMS communication); assessed by the potential participant’s inability to read and/or comprehend the informed consent form.
3. The inability to understand and speak English or French (voice phone call communication); assessed by the potential participant’s replies to the questions, “What is your name?”, “How old are you?”, and “Where do you live?”, “How did you spend your last holidays?”, and the potential participant’s ability to have a brief one-on-one discussion about certain aspects of HIV infection.
4. The refusal to receive a SMS or a phone call as follow-up medical appointment reminders; assessed by asking the potential participant directly if he or she wished to receive a reminder via their mobile phone, or not.

### Analysis plan

Data was coded, entered, and analyzed using the Statistical Package for Social Science (SPSS) version 20.0 for Windows (IBM Corp. Released 2011. IBM SPSS Statistics for Windows, Version 20.0. Armonk, NY: IBM Corp.). We described continuous variables using means with standard deviations, and binary variables using their frequencies and percentages. The chi-square test or its equivalents was used to compare binary variables. All statistical tests were performed using two-sided tests at a 0.05 level of significance. We compared the obstacles between study sites, and between communication by SMS and phone call. The Benjamini-Hochberg method was used to adjust the level of significance of comparison between obstacles in each site [28]. WinPepi version 11.25 was used to specifically adjust *p* values [29].

## Results

We enrolled 301 subjects: 119, 142, and 40 respectively in rural, semi-urban and urban areas. Table 1 shows the general characteristics of the study population. The mean age of caregivers was 42.9 years (SD 13.4) and 46 caregivers (15.3%) were male. Most of them, 148 (49.2%) had completed a primary level of education.

**Table 1:** **General characteristics and obstacles to the use of mobile phone reminders for mHealth in Cameroon**

	**Rural**	**Semi-urban**	**Urban**	**Total**
	**n = 119**	**n = 142**	**n = 40**	**N = 301**
***General characteristics***				
Children				
- Mean age, years	3.6 (3.8)	2.6 (4.0)	3.0 (3.7)	3.1 (3.9)
- Boys	46 (38.7)	70 (49.3)	27 (67.5)	143 (47.5)
Caregivers				
- Mean age, years	42.2 (12.9)	43.1 (13.7)	43.8 (13.8)	42.9 (13.4)
- Men	22 (18.5)	19 (13.4)	5 (12.5)	46 (15.3)
Caregivers level of education				
- No formal	34 (28.6)	39 (27.5)	8 (20.0)	81 (26.9)
- Primary	55 ( 46.2)	67 (47.2)	26 (65.0)	148 (49.2)
- Secondary	16 (13.4)	23 (16.2)	4 (10.0)	43 (14.3)
- University	14 (11.8)	13 (9.2)	2 (5.0)	29 (9.6)
Time to scheduled appointment, days	32.1 (17.7)	27.7 (12.5)	27.8 (15.9)	29.5 (15.3)
***Obstacles***				
At least one obstacle	47 (39.5)	9 (6.3)	3 (7.5)	59 (19.6)
Without mobile phone	14 (11.8)	1 (0.7)	0	15 (5.0)
Unable to communicate via text message in NOL	41 (34.5)	5 (3.5)	1 (2.5)	47 (15.6)
Unable to communicate via voice phone call in NOL	27 (22.7)	4 (2.8)	0	31 (10.3)
Declined to receive text message	3 (2.5)	5 (3.5)	3 (7.5)	11 (3.7)
Declined to receive voice phone call	0	2 (1.4)	1 (2.5)	3 (1.0)

NOL: National official languages.

Data are n (%) or mean (standard deviation).

This study revealed that 80.1% of the study population did not present any of the obstacles to receiving mobile phone reminders. Regarding each study site, the distribution of the absence of obstacles was: 60.5% in rural, 93.7% in semi – urban, and 92.5% in urban settings. The greatest obstacle was the inability to read an SMS message (15.6%) followed by the inability to communicate orally (10.3%) in NOL. Very few caregivers refused to receive a SMS (3.7%) or a phone call (1.0%) to remind them of the child’s upcoming medical appointment. The extent of non-possession of a mobile phone was also low (5.0%) (Table 1).

The occurrence of at least one obstacle to mobile reminders was more frequent in rural than in semi-urban (*p* <0.001) and urban (*p* <0.001) areas. Caregivers without a mobile phone were more common in rural than in semi-urban (*p* <0.001) and urban (*p* = 0.03) areas. The inability to use a NOL for text messaging was more prevalent among caregivers living in a rural area as compared to caregivers living in semi-urban (*p* <0.001) and urban (*p* = 0.002) areas. There were no differences between geographic areas regarding the refusal to receive text messaging reminder and voice phone call reminders. Also, there was no difference between urban and semi-urban areas regarding the mHealth impediments we evaluated (Table 2).

**Table 2:** **Comparison of impediments to mobile phone reminders for mHealth between sites (** ***p*** **values) in Cameroon**

**Obstacles**	**Urban vs. semi-urban**	**Urban vs. rural**	**Semi-urban vs. rural**
At least one obstacle	.73	< .001	< .001
Without mobile phone	1.0	.03	< .001
Unable to communicate via text message in NOL	1.0	< .001	< .001
Unable to communicate via voice phone call in NOL	.58	.002	< .001
Reject to receive text message	.57	.51	.73
Reject to receive voice phone call	.53	.06	.75

NOL: National official languages.

Non-ownership of a mobile phone was associated with geographic areas of residence (*p* <0.001), and with the inability to use a NOL for text messaging (*p* <0.001) and voice phone calling (*p* <0.001) (Table 3). There was no association between caregiver age, sex, level of education attained, or time until the scheduled appointment and the refusal to receive appointment reminder by text message or voice phone call (Table 4). Impediments to using SMS were not significantly different than those to using voice phone calls (Table 5).

**Table 3:** **Comparison of adult caregivers of children requiring follow-up medical care for HIV with and without mobile phone**

	**With phone (n = 286)**	**Without mobile phone (n = 15)**	***p*** **value**
Sites			
- Rural	105 (36.7)	14 (93.3)	< .001
- Semi-urban	141 (49.3)	1 (6.7)	
- Urban	40 (14.0)	0	
Caregivers mean age, years	42.6 (13.5)	46.9 (11.5)	.23
Caregivers male	43 (15.0)	3 (20.0)	.71
Caregivers level of education			
- No formal/Primary	219 (76.6)	10 (66.7)	.36
- Secondary/University	67 (23.4)	5 (33.3)	
Unable to communicate via text message in NOL	38 (13.3)	9 (60.0)	< .001
Unable to communicate via voice phone call in NOL	24 (8.4)	7 (46.7)	< .001
Declined to receive text message	9 (3.1)	2 (13.3)	.09
Declined to receive voice phone call	3 (1.0)	0	1.0

NOL: National official languages.

Data are n (%) or mean (standard deviation).

**Table 4:** **Comparison between adult caregivers who rejected or adhered to SMS/voice phone call reminders**

	**SMS reminder**			**Voice call reminder**			**One of both reminder**		
	**Adhere (n = 290)**	**Reject (n = 11)**	***p***	**Adhere (n = 298)**	**Reject (n = 3)**	***p***	**Adhere (n = 287)**	**Reject (n = 14)**	***p***
Caregivers’ age, years	50.0 (11.9)	42.6 (13.4)	.07	42.9 (13.4)	38.3 (11.5)	.557	47.5 (12.4)	42.6 (13.4)	.18
Male caregivers	42 (14.5)	4 (36.4)	.07	46 (15.4)	0	1.0	42 (14.6)	14 (100.0)	.24
Caregivers’ level of education									
- No formal/Primary	221 (76.2)	8 (72.7)	.73	226 (75.8)	3 (100.0)	1.0	218 (76.0)	11 (78.6)	1.0
- Secondary/University	69 (23.8)	3 (27.3)		72 (24.2)	0		69 (24.0)	3 (21.4)	
Time to scheduled appointment	29.4 (15.4)	31.8 (14.5)	.60	29.5 (15.4)	28.3 (3.5)	.90	29.4 (15.4)	31.1 (12.9)	.69

Data are n (%) or mean (standard deviation).

**Table 5:** **Comparison of impediments to the use of text message and phone call as appointment reminders**

**Obstacles, n (%)**	**Text message**	**Voice phone call**	***p*** **value**
	**n = 301**	**n = 301**	
Refusal	11 (3.7)	3 (1.0)	.054
Unable to communicate	47 (15.6)	31 (10.3)	.052

## Discussion

This study reveals that the use of mobile phones for medical follow-up mHealth appointment reminders in pediatric HIV could potentially apply to 80% of the overall population in Cameroon. Considering each study site separately, the potential penetration of such mHealth use would be different, as we captured 60.5% of caregivers in rural, 93.7% of caregivers in semi - urban and 92.5% of caregivers in urban areas. The greatest obstacle to mobile phone reminders was an adult caregiver’s inability to read an SMS message, followed an inability to communicate orally in English or French, which are Cameroon’s two national official languages. Very few subjects refused to receive a SMS or a phone call to remind them of the child’s medical appointment. The rate of mobile phone non-possession was also low. All impediments to mobile reminders were more frequent in the rural setting, except for the refusal to receive SMS or phone call. SMS or phone call showed no difference in their difficulty of use.

Language illiteracy was the major barrier in our study, as in others [24], and was more pronounced in rural areas in our study. In Uganda, in a region where 80% of people live in rural areas, most persons speak a local language different than NOL [30]. Before SMS and phone calls can be widely implemented as medical appointment reminders, it will be necessary to assess the feasibility of a particular linguistic communication program specific to each health care setting. The literacy rate will need to be improved, especially in rural areas, to help achieve gains in globalization and automation of medical appointment reminders via SMS and phone calls. Health care providers could also adapt and use local languages and dialects to deliver messages about upcoming medical appointments. We suggest that medical assistants in each HIV care center be trained to communicate in the most widely spoken local language (or dialect) in their catchment area in order to reach and include patients and caregivers who communicate exclusively in their local language. The challenge lies in the great number of ethno-linguistic groups in Cameroon, recently assessed at 286 [31]. It will be therefore necessary to target the most widely spoken local languages in each health district.

In our study, 95% of subjects owned a mobile phone. The rate of ownership is higher than that found in Durban, South Africa in 2010 where 81% of people living with HIV had a mobile phone [26] and in Nigeria where 68% of a diabetes population owned a mobile phone [32]. The finding is explained by the exponential increase in the number of subscriptions to mobile phone companies each year [7,8,24]. Mobile phone ownership in our 2013 study is however greater than that found in Kenya in the same year (61.2%) [33]. The difference may reflect the greater proportion of the population living below the national poverty line in Kenya (45.9% in 2005) as compared to Cameroon (39.9% in 2007), and by Cameroon’s higher per capita Gross National Income as compared to Kenya’s [34,35]. In our study, subjects in urban and semi-urban areas owned more mobile phones compared to Kenya’s rural areas [33], due to the fact that the socioeconomic level is lower in rural areas [36]. The observed regional and socioeconomic heterogeneity of mobile phone ownership was also demonstrated in Kenya [37] where factors like gender, educational level, literacy and income are also thought to have an influence on mobile phone ownership [37]. The present study also reveals that people unable to communicate in NOL by text message or orally are most likely not to own a mobile phone. In contrast to another study from sub-Saharan Africa, we found that neither gender nor educational level was a factor in mobile phone ownership [37]. This implies that increasing the NOL literacy rate could lead to an increase in the rate of mobile phone ownership. Although it might be of interest to assess the feasibility and acceptability of using a shared or borrowed mobile phone for appointment reminders and delivery of other healthcare related information for caregivers not owning a mobile phone, we believe that such a “solution” carries significant ethical concerns, especially in regards to privacy and confidentiality.

The acceptability of mobile phones for our mHealth reminder intervention is high (95%) in our study, as it is in other studies in sub-Saharan Africa [24–26,30,38]. In our study, age, sex, and educational level of the caregivers did not influence acceptability. Also, the interval of time until the scheduled follow-up appointment did not influence acceptability. The adult caregivers were agreeable to receiving an appointment reminder by mobile phone independently of how close or far away the upcoming appointment was in time. The willingness of an adult caregiver to receive mobile phone reminders for a child’s follow-up HIV care likely reflects an interest in the well-being and continued optimal care of the child and incorporates the perception that a reminder helps ensure better monitoring and delivery of required ongoing care. It will thus be informative and relevant in future studies to investigate the motivations of caregivers of children exposed to or infected with HIV who refuse to receive mobile phone reminders.

Our study found no significant differences in the rates of refusal between SMS and voice phone calls. Crankshaw et al. found a 99% acceptability for phone calls and 96% for SMS [26]. Their result is very similar to ours; we achieved 99% acceptability for calls and 96.3% for SMS. This suggests that we could freely choose to use SMS or phone calls in sub-Saharan Africa in terms of communication difficulty.

Our study has some limitations. Barriers in addition to those we assessed may impede the use of mobile phones as a medical appointment reminder aid. Examples of obstacles that we did not examine include the timing of sending messages, the unavailability and fluctuations of the wireless network, the phone being powered off, low motivation of medical assistants, and privacy concerns about health [19,24,26,39].

## Conclusions

Despite the proven benefits of mobile health in HIV in sub-Saharan Africa [3,5], the use of mobile phone SMS or voice calls to caregivers providing scheduled appointment reminders for pediatric HIV care in Cameroon encountered a relatively high proportion of obstacles. The most important obstacle was the inability to use spoken or written NOL, followed by non-ownership of a mobile phone. These impediments were higher in a rural area than in urban or semi-urban areas. Rural areas, during the implementation of any health policy based on SMS and phone calls, would thus benefit from a targeted program addressing difficulties in communicating in verbal and written NOL and low mobile phone ownership rate. In terms of acceptability and efficiency, both SMS and phone calls were equivalent when used as mHealth reminder tools in our Cameroonian population of adult caregivers of children requiring HIV care.

## Abbreviations

HIV: Human immunodeficiency virus
MHealth: Mobile health
NOL: National official languages
SMS: Text message (short message service)
